# Significance of Relative Position of Cellulases in Designer Cellulosomes for Optimized Cellulolysis

**DOI:** 10.1371/journal.pone.0127326

**Published:** 2015-05-29

**Authors:** Johanna Stern, Amaranta Kahn, Yael Vazana, Melina Shamshoum, Sarah Moraïs, Raphael Lamed, Edward A. Bayer

**Affiliations:** 1 Department of Biological Chemistry, The Weizmann Institute of Science, Rehovot, Israel; 2 Department of Molecular Microbiology and Biotechnology, Tel Aviv University, Ramat Aviv 69978, Israel; University of Wisconsin, Food Research Institute, UNITED STATES

## Abstract

Degradation of cellulose is of major interest in the quest for alternative sources of renewable energy, for its positive effects on environment and ecology, and for use in advanced biotechnological applications. Due to its microcrystalline organization, celluose is extremely difficult to degrade, although numerous microbes have evolved that produce the appropriate enzymes. The most efficient known natural cellulolytic system is produced by anaerobic bacteria, such as *C*. *thermocellum*, that possess a multi-enzymatic complex termed the cellulosome. Our laboratory has devised and developed the designer cellulosome concept, which consists of chimaeric scaffoldins for controlled incorporation of recombinant polysaccharide-degrading enzymes. Recently, we reported the creation of a combinatorial library of four cellulosomal modules comprising a basic chimaeric scaffoldin, i.e., a CBM and 3 divergent cohesin modules. Here, we employed selected members of this library to determine whether the position of defined cellulolytic enzymes is important for optimized degradation of a microcrystalline cellulosic substrate. For this purpose, 10 chimaeric scaffoldins were used for incorporation of three recombinant *Thermobifida fusca* enzymes: the processive endoglucanase Cel9A, endoglucanase Cel5A and exoglucanase Cel48A. In addition, we examined whether the characteristic properties of the *T*. *fusca* enzymes as designer cellulosome components are unique to this bacterium by replacing them with parallel enzymes from *Clostridium thermocellum*. The results support the contention that for a given set of cellulosomal enzymes, their relative position within a scaffoldin can be critical for optimal degradation of microcrystaline cellulosic substrates.

## Introduction

The cellulosome was first demonstrated in the anaerobic thermophilic cellulolytic bacterium, *Clostridium thermocellum* [1–3] and comprises an extracellular multi-enzymatic complex which efficiently degrades cellulose, the major component of plant cell wall. Its architecture is dictated by a primary scaffoldin subunit, consisting of repeating units of cohesin modules that interact with high-affinity with dockerin-containing enzymes. In addition, a carbohydrate-binding module (CBM) allows targeting to the cellulosic substrate [4, 5]. The cellulosome is attached to the bacterial cell surface via another type of cohesin-dockerin interaction between the primary scaffoldin and an anchoring scaffoldin, which connects to the cell via an S-layer homology (SLH) module [6]. This molecular “machine” results in an organization of the enzymes in close proximity that facilitates stronger synergism among the catalytic units but also minimizes diffusion loss of hydrolytic products for the bacterial host [7]. In the native cellulosome, the scaffoldin possesses multiple copies of cohesins of the same specificity, and the dockerin-bearing enzymes will thus bind randomly into the scaffoldin subunit. Cohesins from different bacteria usually display intraspecies fidelity, although in some cases cross-specificity has been observed among various species [8].

Designer cellulosomes are artificial nanodevices composed of chimaeric scaffoldin and enzymes with cohesins and dockerins of divergent specificities [9]. Owing to the modular structure of the various cellulosomal protein components, we can dismantle and rearrange them into novel types of interacting parts, thereby creating new varieties of cellulosome-like assemblies. By integrating divergent pairs cohesin-dockerin specificities, these original types of cellulosomal structures can provide controlled incorporation of the enzymatic subunits which cannot be achieved using native cellulosomes [10].

Recently, we reported the construction of an extensive combinatorial chimaeric scaffoldin library consisting of trivalent scaffoldins in order to address the question of whether enzyme position within the cellulosome complex contributes to cellulosome performance [11]. Enzymes originating from *C*. *thermocellum* were employed and revealed no significance for the location of the three enzymes tested into the chimaeric scaffoldin.

The designer cellulosome concept was also employed by our group in order to convert the free cellulolytic system of *T*. *fusca* into the cellulosomal form [12–15]. The enzymes were therefore converted to the cellulosomal mode by replacing their inherent CBM with a dockerin module. In this set of publications, we examined the ability of 4 different *T*. *fusca* cellulases, Cel6B, Cel6A, Cel48A and Cel5A, to properly function into designer structures.

However, the most efficient cellulase of the *T*. *fusca* system, the processive endoglucanase Cel9A [16], was never reported to be part of a designer cellulosomal combination. It was only reported that the recombinant cellulosomal form of *T*. *fusca* Cel9A provided higher enzymatic activity than the wild-type form when complexed with a monovalent scaffoldin (i.e., a single cohesin and a CBM) [17].

In preliminary unpublished work performed in our lab [18] the converted cellulosomal form of Cel9A was integrated, together with 2 different recombinant *T*. *fusca* enzymes, into a designer cellulosome complex but the enzyme appeared to be unsuitable for synergistic action with the other enzymes used in this study. Interestingly, Cel9A was always positioned in the middle of the chimaeric scaffoldin. Since this enzyme is relatively large, one possible explanation for the observed poor enzymatic performance in the cellulosome mode may be due to potential steric disturbances.

In the current study, we thus decided to employ the previously described library of chimaeric scaffoldins to test the importance of the position of the recombinant processive endoglucanase Cel9A from *T*. *fusca* relative to two additional enzymatic partners: the recombinant cellulosomal forms of the exoglucanase Cel48A and the endoglucanase Cel5A. These were previously reported to be efficient as designer cellulosome components [13, 14]. In addition, similar enzymes from the bacterial system of *C*. *cellulolyticum*, namely Cel9G, Cel48F and Cel5A, provided high synergistic effect into designer cellulosome [19]. In order to further validate our hypothesis, similar recombinant enzymes were selected from the cellulolytic system of *C*. *thermocellum* (Cel9R, Cel48S and Cel5G) and integrated into the same chimaeric scaffoldin library.

## Materials and Methods

### Cloning

Plasmids encoding for the trivalent scaffoldin library were prepared as described earlier [11]. Plasmids encoding for monovalent scaffoldins Scaf**·** ***c***A, Scaf**·** ***c***B and Scaf**·** ***c***T were produced as in an earlier publication [8].

*T*. *fusca* wild-type enzymes Cel48A and Cel5A were cloned as in previous work [20]. Chimaeric forms of *T*. *fusca* enzymes *b*-48A and 5A-*t* were produced as reported previously [13, 14]. However, the recombinant dockerin form *a*-9A was constructed using the following primers: 5'AATT**GGTACC**CCCCTTGGCCACGGGA ACCG 3' and 5' TATT**CTCGAG**TTA TTCTCCGCCGCCGGGCTCTTCC 3' to amplify the catalytic module from *T*. *fusca* genomic DNA (*KpnI* and *XhoI* sites are in boldface type) and 5' GCCA**CCATGG**CCC ACCATCACCATCACCATAAATTTATATATGGT GATGTTGATGG 3' and 5' TATC **GGTACC**TTCTTCTTTCTCTTCAACAGGG 3' to amplify the dockerin of ScaB from *A*. *cellulolyticus* genomic DNA (*NcoI* and *KpnI* sites are in boldface type).

*C*. *thermocellum* wild-type enzyme Cel5G was amplified from genomic DNA using 5'CGTC**TCATGA**CCGCCGTCGACAGCAACAACGA3' and 5'GGAG**CTCGAG**GTG GTGTGCGGCAGTTTG3' (*BspHI and XhoI* sites are in boldface type). The recombinant dockerin forms 9R-*a* and 48S-*b* were constructed using the following primers: 5'AGCA **CCATGG**CAGACTATAACTTGGAGAA 3' and 5'AGCA**GGATCC**GTACCATTG GGTTCTAC ACC 3' (*NcoI* and *BamHI* sites are in boldface type); 5' AATCG**CCATGG** GCCCTACA AAGG CACCTACAAAAG 3' and 5' GAATC**AGATCT**TTATATGTC ATATCCGG GAAGTATG 3' (*NcoI* and *BglII* sites are in boldface type) for the catalytic modules of Cel9R and Cel48S respectively from the genomic DNA of *C*. *thermocellum*. The dockerin of ScaB from *A*. *cellulolyticus* and the dockerin of ScaA from *B*. *cellulosolvens* were amplified using the following primers: 5' TACG**GGATCC**CAA ATTTATATATGGTGATGTTG 3' and 5'AATC**CTCGAG**TTCTTTCTCTTCAA CAGGG 3'; 5' TCACCA**GGATCC**TCCAAAAGGCACAGCTACAGT 3' and 5' GTG GCC**CTCGAG**CGCTTTTTGTTCTGCTGGGAA 3' (*NcoI* and *BamHI* sites are in boldface type).

The different modules were assembled in the linearized pET28a plasmid.

All enzyme constructs were designed to contain a His tag for subsequent purification. PCRs were performed using ABgene Reddymix x2 (Advanced Biotechnologies Ltd., United Kingdom), and DNA samples were purified using a HiYield gel/PCR fragment extraction kit (Real Biotech Corporation, RBC, Taiwan).

### Protein expression and purification

The nine trivalent chimaeric scaffoldins from the library were purified as reported previously [11] as well as the monovalent scaffoldins Scaf**·** ***c***A, Scaf**·** ***c***B and Scaf**·** ***c***T proteins were produced as in an earlier publication [8].

*T*. *fusca* wild-type enzymes Cel48A and Cel5A were purified as previously reported [20] whereas the wild-type Cel9A was a generous gift from the group of David Wilson (Cornell University, New York).

The *C*. *thermocellum* wild-type enzyme Cel5G and the chimaeric forms 9R-*a* and 48S-*b* were produced by expression of relevant plasmids into *E*. *coli* BL21 (lDE3) pLysS cells. The proteins were extracted and purified on an Ni-nitrilotriacetic acid (NTA) column (Qiagen, Redwood city, USA), as reported earlier [15].

Purity of the recombinant proteins was assessed by SDS-PAGE on 12% acrylamide gels, and the fractions containing the pure recombinant protein were pooled and concentrated using Amicon Ultra 15 ml 50,000 MWCO concentrators (Millipore, Bedford, MA, USA). Protein concentration was estimated from the absorbance at 280 nm based on the known amino acid composition of the protein using the Protparam tool (http://www.expasy.org/tools/protparam.html). Proteins were stored in 50% (vol/vol) glycerol at –20°C.

### Affinity-based ELISA

The matching fusion-protein procedure of Barak et al. [21] was followed to determine cohesin-dockerin specificity of interaction.

### Non-denaturating PAGE

To check the extent of interaction and determine exact equimolar ratios between the cohesin-bearing scaffoldin and dockerin-bearing enzymes, a differential mobility assay on non-denaturing PAGE gels was used. Protein samples (4 to 8 μg each) were added to Tris-buffered saline (TBS) (pH 7.4) supplemented with 10 mM CaCl_2_ and 0.05% Tween 20 to a total volume of 30 μl. The tubes were incubated for 2 h at 37°C. Sample buffer (7.5 μl in the absence of SDS) was added to 15 μl of the reaction mixture, and the samples were loaded onto non-denaturating gels (4.3% stacking gels/9% separating gels).

### Affinity pull-down assays

Equimolar amount of pure proteins were prepared (100 picomoles each in 50 mM Acetate buffer [pH 5.0], 12 mM CaCl2, 2 mM EDTA) and incubated for 2 hours at 37°C in the presence of 10% cellobiose (Sigma-Aldrich Chemical Co, St. Louis, MO, USA). Cellobiose is known to bind to the catalytic module of the enzymes and then blocks partial binding of the enzymes to the cellulosic substrate. The fractions were then gently mixed with microcrystalline cellulose (Avicel, FMC Biopolymer Philadelphia, PA) for 1 hour. The tubes were then centrifuged at 16,000 × g for 2 min. The supernatants (containing unbound proteins) were carefully removed and supplied with SDS-containing buffer to a final volume of 60 μl. The pellets (containing bound proteins) was washed twice by resuspension in 200 μl of 50 mM Acetate buffer supplemented with 0.05% Tween 20 to eliminate nonspecific binding. It was then centrifuged at 16,000 × g for 2 min and resuspended in 60 μl of SDS-containing buffer. Both resultant unbound and bound fractions were boiled for 10 min and then analyzed by SDS-PAGE using a 10% polyacrylamide gel.

### Enzymatic activity

Degradation of the crystalline cellulosic substrate Avicel was assayed using specified enzyme concentrations. For enzymes bound to scaffoldins, the various components were introduced in equimolar ratios into a Tris-buffered saline (pH 7.4) supplemented with 10 mM CaCl_2_ and 0.05% Tween 20 to a total volume of 30 μl and were allowed to interact for 2 h at 37°C. The suspensions were then incubated at 50°C for 24–72 h, in the presence of 80 μl of 10% (w/v) Avicel in a final volume of 300 μl (50 mM acetate buffer, pH 5.0, 12 mM CaCl_2_, 2 mM EDTA). Samples were shaken for the duration of the incubation. Reactions were terminated by immersing the sample tubes in ice water, after which the samples were centrifuged at maximum speed to remove the substrate. Dinitrosalicylic acid (DNS) solution (150 μl) was added to 100 μl supernatant fiuids [22], and the reaction mixture was boiled for 10 min. Optical density was measured at 540 nm, and activity was determined from a glucose standard curve. The blank was determined as the optical density obtained by an enzyme-free control. All assays were performed in triplicate.

## Results

### Construction and expression of recombinant proteins

**Fig 1:**
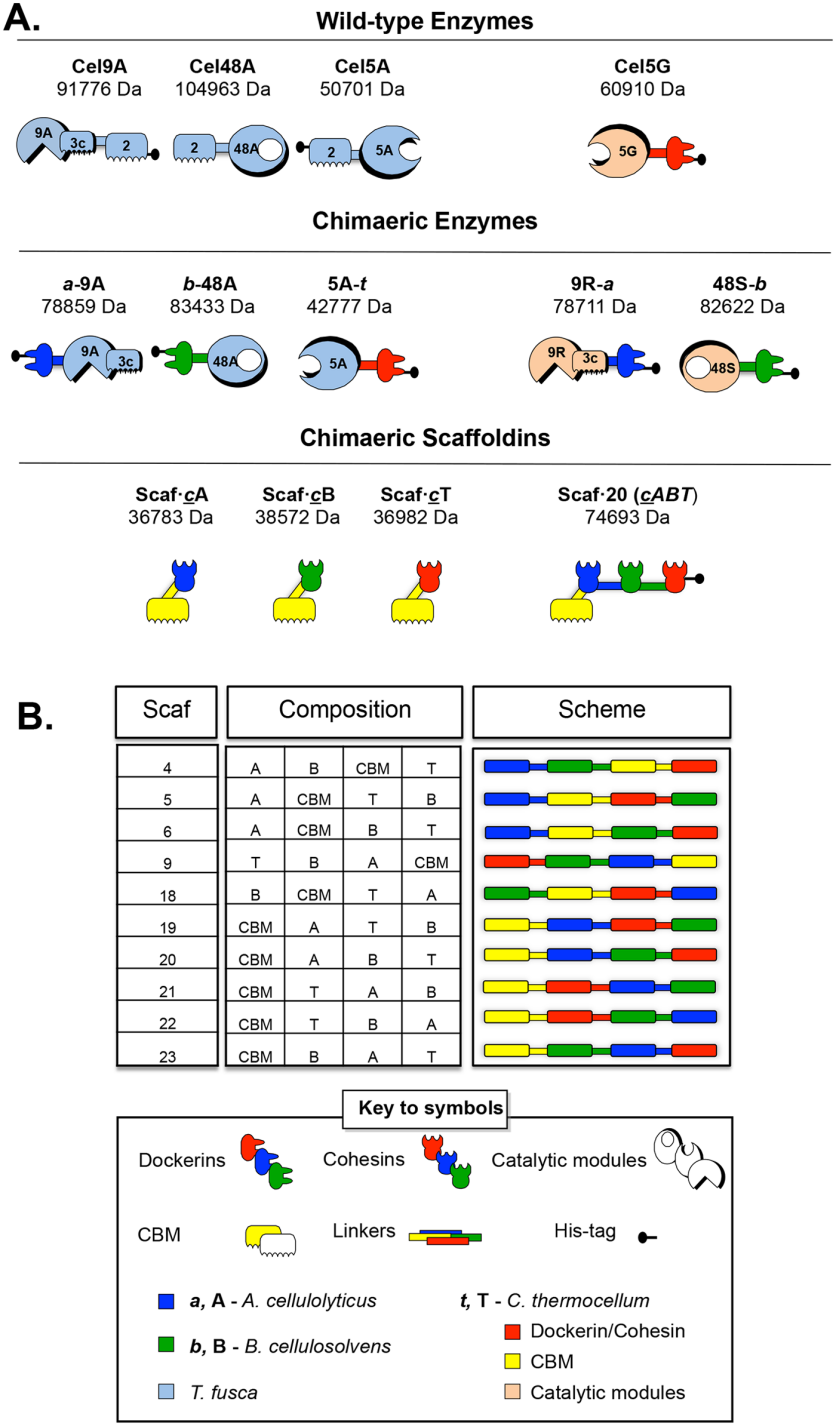
Schematic representation of the recombinant proteins used in this study. The bacterial origin of the representative modules is indicated as follows: dark blue, *A*. *cellulolyticus*; green, *B*. *cellulosolvens*; powder blue, *T*. *fusca*; red, yellow and peach for *C*. *thermocellum* dockerin, CBM and catalytic modules, respectively. In the shorthand notation for the recombinant enzymes, the numbers 9, 48 and 5 correspond to the GH family (GH9, GH48 and GH5) of the catalytic modules; uppercase characters (A, B and T: representing *A*. *cellulolyticus*, *B*. *cellulosolvens* and *C*. *thermocellum*, respectively) indicate the bacterial origin of the cohesin modules, and lowercase (*a*, *b* and *t*) indicate the origin of the dockerin modules. The *T*. *fusca* enzymes each contains a cellulose-specific family 2 CBM, and that of the chimaeric scaffoldins are derived from the family 3a CBM from *C*. *thermocellum* CipA. The *C*. *thermocellum* Cel9R processive endoglucanase is attended by a specialized family 3c CBM, which is intimately linked to the structure and function of the enzyme. The table embedded in the figure provides a schematic color-coded representation of the nine chimaeric scaffoldins employed in this study. The left column indicates the number of the scaffoldin corresponding to their modular composition: CBM and divergent cohesin modules [11].

The schematic representation of the enzymes and scaffoldins used in this study are represented in Fig 1.

The creation of the recombinant forms (Fig 1A) of Cel48A and Cel5A was achieved as previously reported [13, 14]. The recombinant form of Cel9A named *a*-9A was obtained by removing the CBM2 of the wild type at the C-terminus and by adding a dockerin module from *A*. *cellulolyticus* (from ScaB) at the N-terminus. Initially, we tried to construct a 9A-*a* chimaera, where the *A*. *cellulolyticus* dockerin is positioned at the C-terminus of the enzyme (downstream to the catalytic module) to coincide with the original position of the wild-type enzyme's CBM2. This policy was followed with most of the previously designed *T*. *fusca* enzymes [23]. Although the DNA construction was successful, we could not obtain a purified sample of the chimaeric protein although various methods of purification were attempted. To circumvent this problem, we decided to construct the *a*-9A chimaera (with the dockerin on the “reversed” side of the catalytic module), which was easily purified. Indeed, we based our decision on the results obtained for the 5A-*t* chimaera (reversed chimaera), that was shown to be even more efficient than the conventional strategy [13].

The recombinant forms of the processive endoglucanase Cel9R and the endoglucanase Cel48S from *C*. *thermocellum* were obtained by replacing their native dockerins by a dockerin module of *A*. *cellulolyticus* (from ScaB) and *B*. *cellulosolvens* (from ScaA), respectively. The dockerin modules were chosen to fit with the corresponding dockerin modules of *T*. *fusca* chimaeric enzymes from the same family (i.e., the endoglucanase Cel5G is employed in the wild-type form, since it corresponds to the *T*. *fusca* Cel5A enzyme that was converted to the cellulosomal mode by adding a *C*. *thermocellum* dockerin).

Monovalent scaffoldins Scaf**·** ***c***A, Scaf**·** ***c***B and Scaf**·** ***c***T, comprising only a single cohesin (with specified specificity) and a CBM3 from *C*. *thermocellum*, provided analysis of targeting effect alone. They were produced as described previously [8].

The trivalent scaffoldins (Fig 1B) employed in this study were selected from the previously reported library of chimaeric scaffoldins [11]. All of them possess long intermodular linkers, since it was reported that long linkers provide an advantage for enhanced degradation. Ten different chimaeric scaffoldins were selected from the library to check the importance of the relative position of the enzymes and the CBM. Two of these scaffoldins (21 and 23) allow the processive endoglucanase *a*-9A to be in the middle, three scaffoldins (5, 18 and 19) placed the endoglucanase 5A-*t* in the middle, next to *a*-9A, five scaffoldins (4, 6, 9, 20 and 22) integrated the exoglucanase *b*-48A in the middle, separating 5A-*t* from *a*-9A, with a CBM either close to 5A-*t* or to *a*-9A.

All purified native and recombinant proteins exhibited a single major band on SDS-polyacrylamide gels (data not shown) with a mobility pattern consistent with its molecular mass.

### Analysis of protein interactions

The specificities of the dockerins (enzymes) and cohesins (scaffoldins) were examined semi-quantitatively by affinity-based ELISA assays in microtiter plates [21]. Each cohesin/dockerin module was able to bind consistently with their matching partner and showed no or very poor binding to non-matching ones (Supplementary materials: S1 Fig).

The stoichiometric ratios for each relevant protein couple (i.e., each enzyme and its respective scaffoldin) were obtained by performing non-denaturating PAGE assay. Stoichiometric mixtures of the enzymes and the scaffoldin resulted in a single major band with altered mobility (the band became larger and its position shifted; Supplementary materials: S2 Fig).

**Fig 2:**
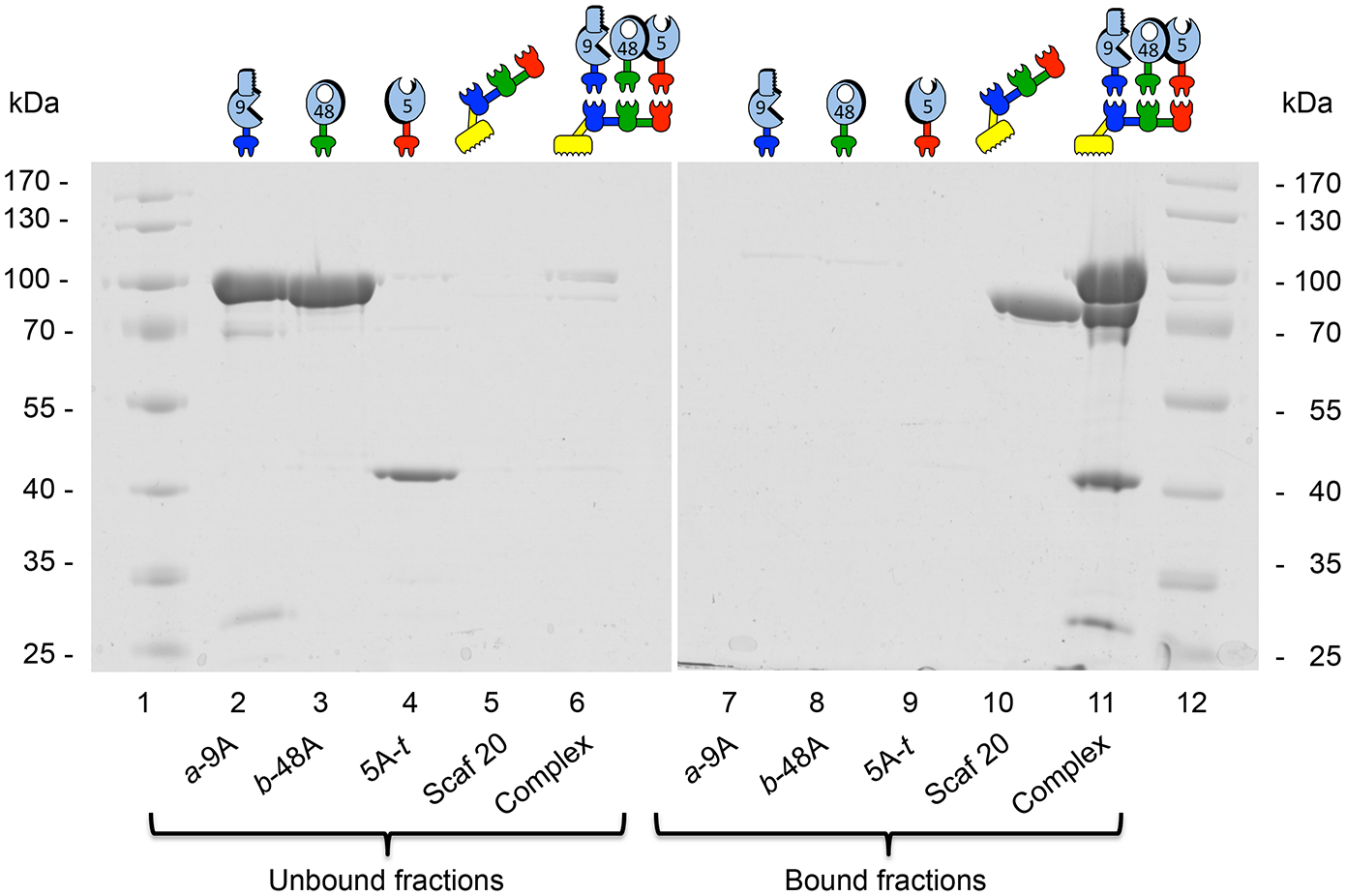
Analysis of complex formation. All chimaeric enzymes and scaffoldins were first assayed individually for binding to a cellulosic substrate. Relevant enzymes and scaffoldin were then mixed together at equimolar ratios and subsequently introduced to a cellulosic substrate (Avicel). The cellulose-binding ability of both individual proteins and the resultant complex was determined by examining the cellulose-bound (lanes 7–11) and unbound (lanes 2–6) fractions by SDS-PAGE. Samples include: lane 1 and 12, molecular weight markers. Lane 2 to lane 6, unbound fractions with the following details: lane 2, *a*-9A; lane 3, *b*-48A; lane 4, 5A-*t*; lane 5, Scaf20; lane 6, Complex of *a*-9A, *b*-48A, 5A-*t* and Scaf20. Lane 7 to lane 11 are bound fractions: lane 7, *a*-9A; lane 8, *b*-48A; lane 9, 5A-*t*; lane 10, Scaf 20; lane 11, Complex of *a*-9A, *b*-48A, 5A-*t* and Scaf20. In the presence of the chimaeric scaffoldin, the enzymatic components were associated with the cellulose-bound fraction; whereas in its absence, they remained in the unbound fraction.

Formation of elaborate complexes (i.e., 3 enzymes and a scaffoldin) was examined by affinity-pull down assay using Avicel (Fig 2). For this purpose, relevant proteins preparations were added to the cellulosic substrate. The bound and unbound fractions were then examined by SDS-PAGE. CBM-lacking proteins i.e. chimaeric enzymes were mostly absent from bound fractions and clearly appeared in the unbound fractions. However, in the presence of the chimaeric scaffoldin, almost no band was present in the unbound fractions, but all components were present in the bound fraction. This demonstrates that the complex was indeed formed at stoichiometric ratios and that it was able to bind strongly to the cellulosic substrate (Supplementary Materials, S3 Fig demonstrates the stability of the trivalent complex, consisting of 3 enzymes and a scaffoldin, even after a 72-h incubation period at 50°C).

### Enzymatic activity of individual *T*. *fusca* enzymes

**Fig 3:**
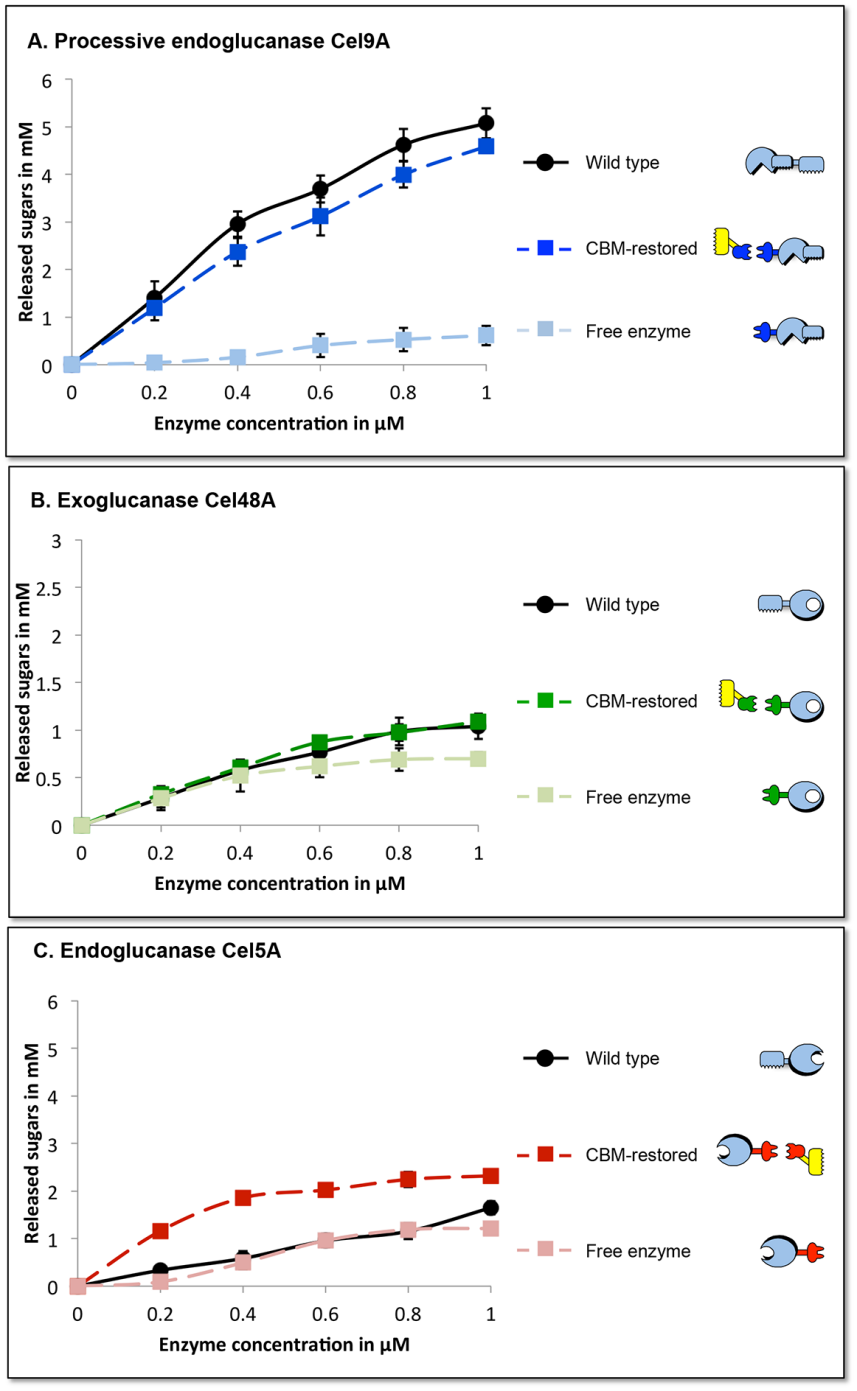
Cellulolytic activity profiles of the three *T*. *fusca* enzymes used in this work. Enzymatic activity on a crystalline cellulosic substrate (Avicel) were determined for the wild-type processive endoglucanase Cel9A, the exoglucanase Cel48A and the endoglucanase Cel5A and for their respective dockerin-bearing chimaeras (*a*-9A, *b*-48A and 5A-*t*) in the presence or absence of matching monovalent scaffoldins. Cellulose degradation is reported as mM total reducing sugars following a 24-h reaction period at 50°C at different enzyme concentrations (ranging from 0 to1 μM).

The recombinant cellulosomal *T*. *fusca* enzymes were tested individually for their ability to function in the designer cellulosome context (Fig 3). Each dockerin-bearing enzyme was assayed either in the free state or bound to a monovalent chimaeric scaffoldin, and the resultant activity on the Avicel substrate was compared to that of the wild-type form.

The cellulosomal chimaeric form of *T*. *fusca* exoglucanase Cel48A and endoglucanase Cel5A were employed in previous publications [13, 14]. Here, we consistently observed that when the recombinant cellulosomal form of the enzyme was bound to its respective monovalent scaffoldin (CBM-restored) the activity was equivalent to that of the wild-type for the exoglucanase *b*-48A and even higher for 5A-*t*.

On the other hand, the enzymatic activity of the chimaeric cellulosomal form of the processive endoglucanase *a*-9A has never been tested in the past. In this experiment, we also found that when the recombinant cellulosomal form of the enzyme was bound to its respective monovalent scaffoldin (CBM-restored) the activity was roughly equivalent to that of the wild-type enzyme, thus indicating its suitability for use in the designer cellulosome format.

### Enzymatic activity of designer cellulosomes using recombinant *T*. *fusca* enzymes

#### Effect of enzyme position on overall activity of designer cellulosomes

The three recombinant cellulosomal forms of the *T*. *fusca* enzymes *a*-9A, *b*-48A and 5A-*t* were integrated into various trivalent chimaeric scaffoldins selected from the previously described library [11].

**Fig 4:**
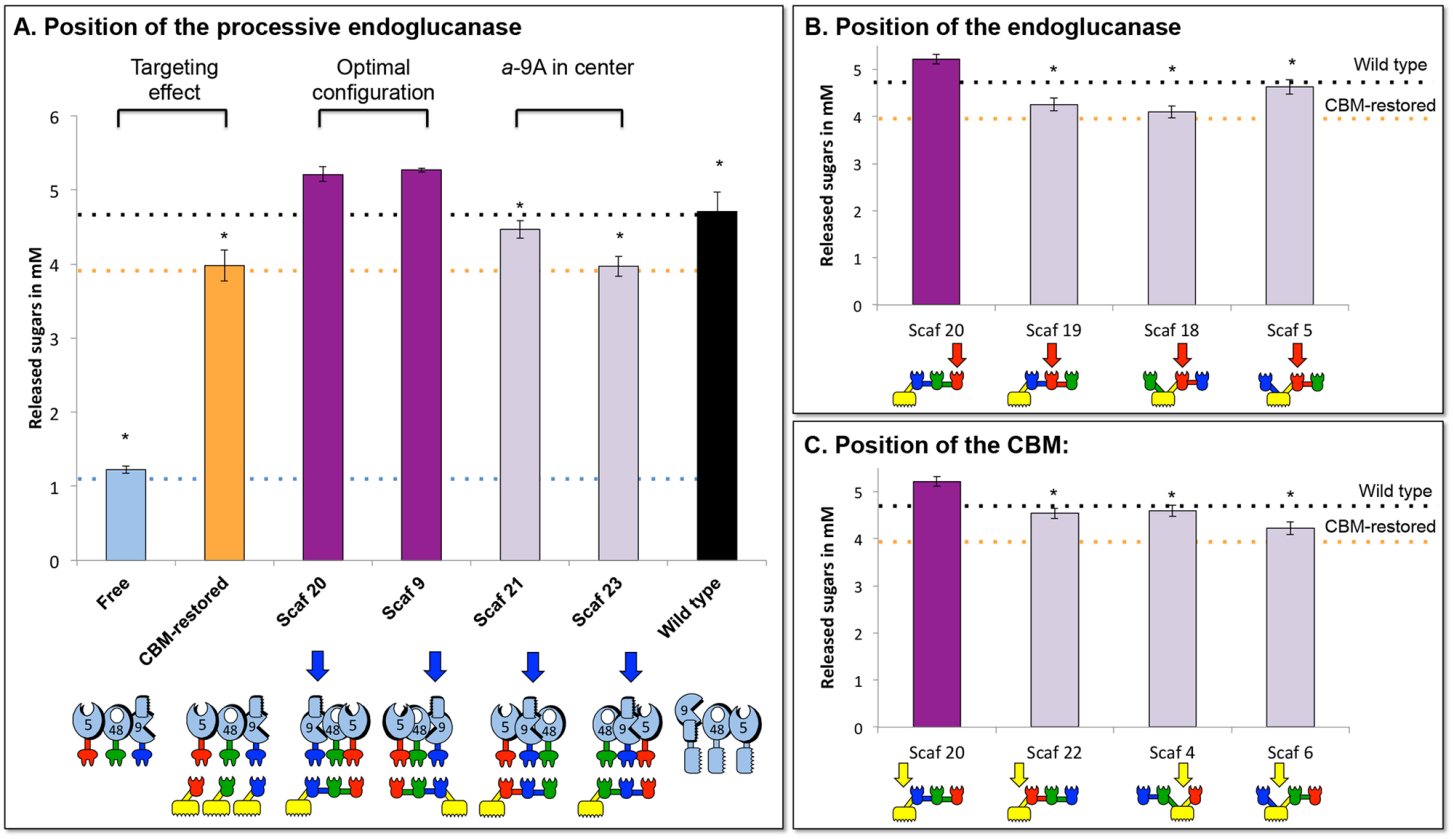
Comparative Avicel degradation by the three recombinant *T*. *fusca* enzymes either (i) in the free state, (ii) bound to corresponding monovalent scaffoldins (CBM-restored state) or (iii) bound to different chimaeric scaffoldins (designer cellulosomes). By varying the modular arrangement of the cohesins in the respective chimaeric scaffoldin, the effect of various designer cellulosome components on enzymatic activity can be examined as follows: A, the influence of the position of the processive endoglucanase (*a*-9A, blue arrows); B, the influence of the position of the endoglucanase (5A-*t*, red arrows); and C, the importance of the position of the CBM (yellow arrows). Enzymatic activity is defined as mM total reducing sugars following a 72-h reaction period at 50°C with each enzyme and scaffoldin concentration fixed at 0.4 μM. Stars indicate a statistically significant difference (lower than) with the two most efficient scaffoldins Scaf 20 and Scaf 9.

Initially, the importance of the position of the processive endoglucanase *a*-9A was tested. Since it is a large enzyme, we suspected possible steric disturbance. For this purpose, we compared the overall activity of the 3 enzymes when the processive endoglucanase was situated either between 2 enzymatic partners (Scaf 21 or Scaf 23) or not (Scaf 20 and Scaf 9 are mirror images of each other) (Fig 4A). Targeting and proximity effects of the different designer cellulosome compositions can be deduced by comparison of the mixture of the three enzymes either in the free state (targeting) or bound to individual monovalent scaffoldins (proximity, see CBM-restored). We observed that only when the processive endoglucanase was not positioned between 2 enzymatic partners (Scaf 20 or Scaf 9), the highest proximity effect was obtained and overall enzymatic activity was enhanced, compared to that of the mixture of the wild-type enzymes. It therefore follows that the recombinant processive endoglucanase employed in this study should not be placed in between two enzymatic partners (Scaf 21 or Scaf 23) for optimal activity of designer cellulosomes.

We then tested the importance of the position of endoglucanase 5A-*t* which is the smallest enzyme in the combination (Fig 4B). When the endoglucanase was placed in the middle of the designer cellulosome (between 2 enzymatic partners for Scaf 19, or between an enzyme and a CBM for Scaf 18 and Scaf 5), the overall enzymatic activity was significantly lower. One possible interpretation of this result is that the proximity between the processive endoglucanase and the endoglucanase causes an unproductive competition between these two functionally similar enzymes. This is supported by the fact that this anti-proximity effect appears to be considerably less important when the CBM module (Scaf 5) is positioned in between the two enzymes. We therefore deduced that the endoglucanase should not be in close proximity to the processive endoglucanase.

We concluded that the optimal order for position of these three enzymes is as follows: processive endoglucanase, exoglucanase and endoglucanase. As a consequence of the enzyme dockerin specificities, the cohesin sequence A-B-T would be preferred for the trivalent scaffoldin.

We then tested the importance of the position of the CBM in the various scaffoldins (Fig 4C). We selected three chimaeric scaffoldins in which the positions of the three enzymes corresponded to the cohesin sequence A-B-T of the scaffoldins but only where the position of the CBM was different. Thus, Scaf 22 placed the CBM at the opposite side of the processive endoglucanase, adjacent to the endoglucanase. Scaf 4 positioned the CBM in the middle of the sequence between the endoglucanase 5A-*t* and the exoglucanase *b*-48A. Both resulted in distal positions of the CBM relative to the processive endoglucanase *a*-9A. For both scaffoldins, we noticed that the overall enzymatic activity was lower. In addition, Scaf 6 placed the CBM in the middle of the sequence between the processive endoglucanase and the exoglucanase. Also here, we observed lower overall enzymatic activity suggesting that the CBM should not be placed in the middle of the scaffoldin.

#### Contribution of enzymatic couples to designer cellulosome-mediated cellulose degradation

**Fig 5:**
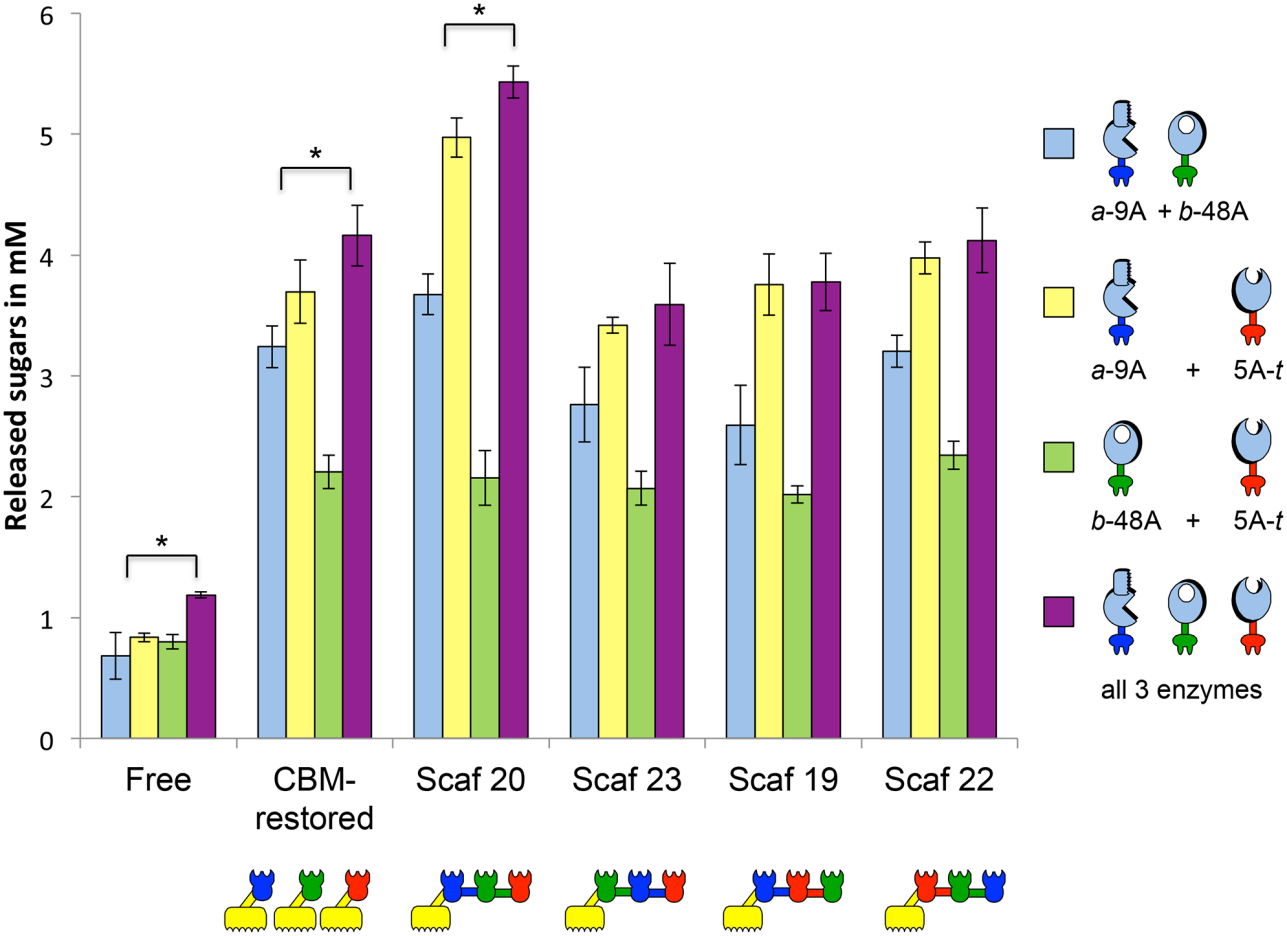
Synergistic activity of all possible combinations of the three *T*. *fusca* recombinant enzymes either (i) in the free state, (ii) bound to respective monovalent scaffoldins (CBM-restored) or (iii) bound to different chimaeric scaffoldins, for degradation of Avicel. Assays were conducted for 72 h, with incubation at 50°C, at an enzymatic concentration of 0.4 μM. Stars indicate a statistically significant difference (lower than) between the couple composed of *a*-9A and 5A-*t* in comparison with the combination of the three enzymes together.

The detailed contribution of each enzymatic couple into chimaeric scaffoldins was examined, in order to obtain additional insight into the importance of enzyme location. All possible combinations of two enzymes were mixed and compared to the overall degradation by the three enzymes together (Fig 5). Only four scaffoldins were employed here, none of them with a CBM in the middle of the cohesin sequence: Scaf 20 which represents the optimal configuration, Scaf 23 which positions the processive endoglucanase between 2 enzymatic partners; Scaf 19, which positions the endoglucanase between 2 enzymatic partners; Scaf 22, which places the CBM at the opposite side of the processive endoglucanase.

Interestingly, the most potent pair of enzymes was found to be the processive endoglucanase and the endoglucanase. In most trivalent scaffoldins (Scaf 23, Scaf 19 and Scaf 22), the level of cellulose degradation produced by this couple was as efficient as the combination of the three enzymes. The observed anti-proximity effects between the processive endoglucanase and the endoglucanase should therefore be avoided. Regarding trivalent chimaeric scaffoldin compositions, only in the optimal configuration of scaffoldin Scaf 20, was further enhanced synergy observed owing to the presence of the exoglucanase.

#### Enzymatic activity of designer cellulosomes using corresponding *C*. *thermocellum* enzymes

We examined whether the characteristic properties of these three types of enzymes as designer cellulosome components are unique to the *T*. *fusca* bacterium or whether the same enzyme types would perform similarly, regardless of their origin. For this purpose, we replaced the *T*. *fusca* enzymes with their closest orthologues from *C*. *thermocellum*. According to percent homology we selected: *C*. *thermocellum* Cel48S to replace *T*. *fusca* exoglucanase Cel48A and Cel9R to replace the Cel9A processive endoglucanase. As we were not able to find a proper percent of homology to *T*. *fusca* Cel5A into *C*. *thermocellum* enzymes, Cel5G was selected since it is a close enzyme in term of activity and is one of the major player in this bacterium [24]. *C*. *thermocellum* enzymes were equipped with dockerin modules that corresponded to those of the *T*. *fusca* orthologue (Fig 1). In this case, the same four scaffoldins described in Fig 5 were employed.

**Fig 6:**
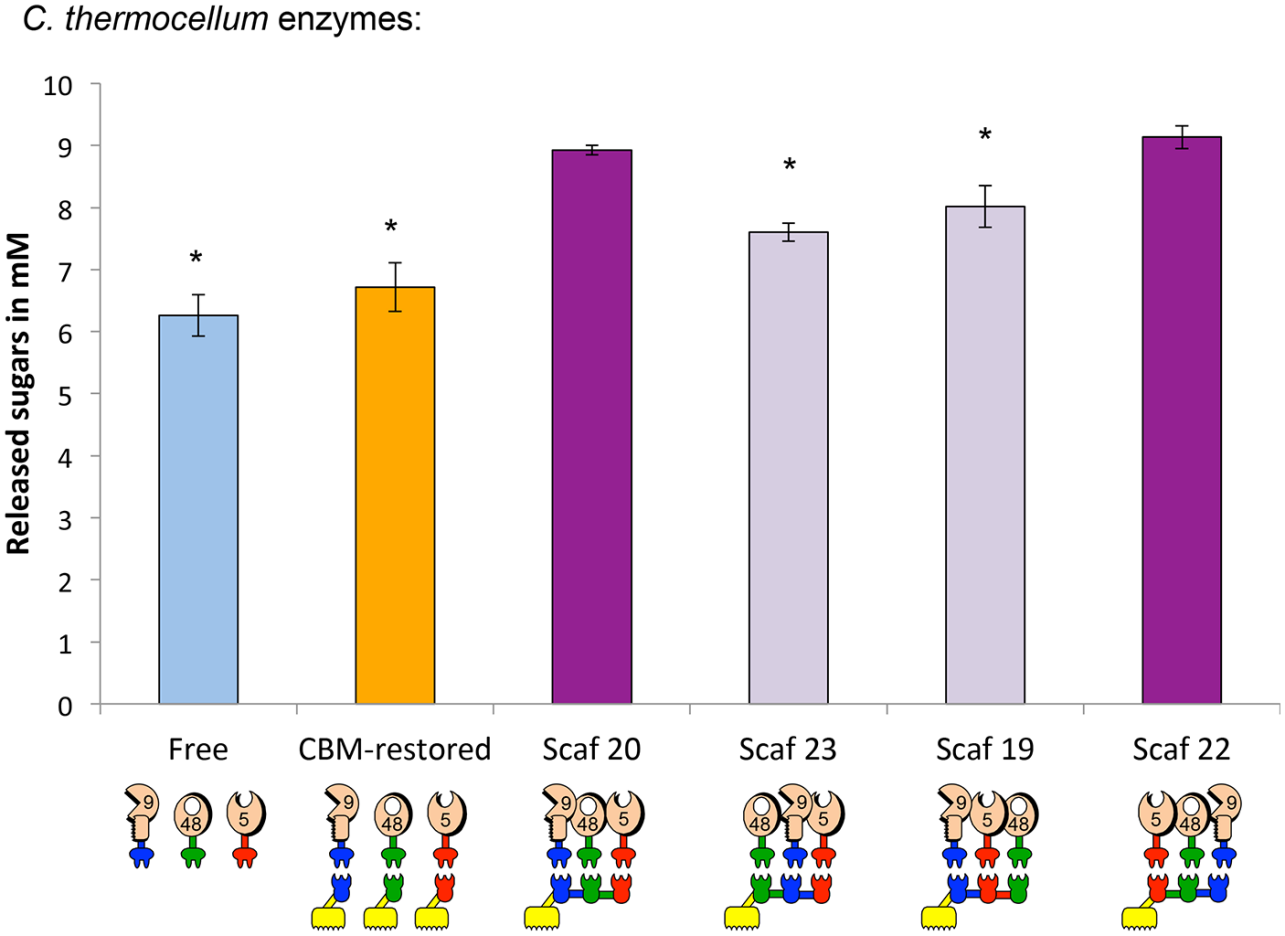
Comparative Avicel degradation by recombinant enzymes from *C*. *thermocellum*, either (i) in the free state, (ii) bound to respective monovalent scaffoldins (CBM-restored) or (iii) bound to different chimaeric scaffoldins. Enzymatic activity is defined as mM total reducing sugars following a 24-h reaction period at 50°C with each enzyme and scaffoldin concentration fixed at 0.4 μM. Stars indicate a statistically significant difference (lower than) with the two most efficient scaffoldins Scaf 20 and Scaf 22.

For improved efficiency of cellulosic substrate degradation, location within a scaffoldin also appeared to be important for the *C*. *thermocellum* enzymes (Fig 6). However, the observed differences were less pronounced than for the recombinant *T*. *fusca* enzymes although still statistically significant. As a rule, the processive endoglucanase 9R-*a* should not be between 2 enzymatic partners (Scaf 23) and not adjacent to the endoglucanase Cel5G (Scaf 19). In this case, the location of the CBM appeared not to be relevant (See Scaf 22).

## Discussion

Previous studies on cellulosome composition suggested that cellulosomes are randomly assembled, principally due to the nearly identical specificities of the scaffoldin-borne cohesins to respective dockerin-bearing enzymes [4, 25]. However, various proteomic studies demonstrate that the cellulosome composition differs according to the substrate [26, 27]. One hypothesis argues that these variations in cellulosomal compositions are controlled by the expression levels of the relevant genes although the precise mechanisms remain unclear [24, 28].

Based on this hypothesis, most studies employing the designer cellulosome strategy accord minimal attention to the position of the enzymes in the chimaeric scaffoldin. In these studies, the cohesin sequences of the chimaeric scaffoldin were prepared in random fashion, although successful combinations were generally observed [20, 23, 29, 30]. Recently, our lab reported a synthetic biology approach in order to analyze the importance of the position of the different modules into trivalent chimaeric scaffoldins [11]. In that study, recombinant enzymes were obtained from the bacterium *C*. *thermocellum*: i.e., the processive endoglucanase Cel9K, the exoglucanase Cel48S and the endoglucanase Cel8A. For this particular set of cellulases, the results do not indicate a preferential arrangement for the chimaeric scaffoldin. Nevertheless, this combinatorial scaffoldin library appears to be a potent tool for further study of other glycoside hydrolase families or from other bacterial organisms.

In the present study, we employed this strategy in order to check if the position of the recombinant processive endoglucanase Cel9A from *T*. *fusca* is important in order to determine optimal levels of cellulose degradation. For this purpose, we examined the degradation of a microcrystalline substrate by combining recombinant cellulosomal forms of Cel9A together with the exoglucanase Cel48A and the endoglucanase Cel5A into selected chimaeric scaffoldins from the combinatorial library. Contrary to the previous observations, here it appears that for this particular set of enzymes their location is indeed critical for optimal degradation of microcrystalline cellulose.

The results indicate that the processive endoglucanase should not be positioned in the middle of the trivalent chimaeric scaffoldin. Two previous studies have also addressed the integration of the enzymes into scaffoldin: both including GH9 enzymes, one from *C*. *thermocellum* [31] and one from *C*. *cellulolyticum* [32]. Both of these studies revealed that these enzymes are more selective than others for binding to the scaffoldin. According to the first study which is a computer simulation, the shape and flexibility of the enzymes are the most important factors to determine their binding to the scaffoldin. The volume, mass and enzyme concentration are irrelevant [31]. On the other hand, the second study comprised an experimental approach, and indicated that the binding of an initial enzyme to a cohesin has a direct influence on the binding of the subsequent enzyme to the next cohesin module [32]. These two mechanisms could possibly explain which strategy the bacterium employs in order to fabricate a potent cellulosome for degradation of cellulosic substrates. It is clear that better understanding of cellulosome assembly will be beneficial for future manufacture of optimized designer cellulosome assemblies.

In addition, our results suggest that the processive endoglucanase should not be in close proximity with the GH5 endoglucanase. Indeed, these two enzymes possess similar types of functionalities, and their immediate proximity may cause unproductive competition between the two catalytic sites. In this context, it is interesting to note that in the previously cited experimental study [32], the processive GH9 endoglucanase do not undergo preferential integration into a mini-scaffoldin together with the GH5 endoglucanase. The relevance of this anti-proximity effect was underscored in the current study by the detailed analysis of degradation by the different enzymatic couples. The results demonstrated that the most potent couple of enzymes was composed of the processive endoglucanase and the endoglucanase. Only a particular configuration of the chimaeric scaffoldin (Scaf20 or Scaf9) did the exoglucanase contribute additional degradation of the substrate.

These two properties of the GH9 processive endoglucanase as a viable designer cellulosome component (location on the scaffoldin and distance from the GH5 endoglucanase) were observed using recombinant enzymes from both *T*. *fusca* and *C*. *thermocellum*. Moreover, in a previous study employing homologous recombinant enzymes from *C*. *cellulolyticum*, a successful combination was obtained using a chimaeric scaffoldin in which the GH9 processive endoglucanase was placed at the beginning of the scaffoldin, at a distance from the GH5 endoglucanase and together with the GH48 exoglucanase [19]. This strongly supports the experimental design in the present work, and the relative position of a GH9 processive endoglucanase should be considered in subsequent design of artificial chimaeric cellulosomes. It also suggests that the importance for enzyme position into a scaffoldin is also related to their functionality in addition to their size or biophysical properties.

The results presented in this work also show that the position of the CBM was important only for recombinant enzymes originating from *T*. *fusca*. In this case, it was important that the processive endoglucanase was placed close to the CBM. Indeed, in contrast to *C*. *thermocellum*, *T*. *fusca* possesses a free enzymatic system where each enzyme bears a CBM. Thus, these enzymes may be less robust within the designer cellulosome context than the native cellulosomal enzymes. Specifically, the processive endoglucanase Cel9A is a complex modular enzyme composed of a catalytic module, a CBM3c module, a fibronectin III-like domain and a CBM2. Various lines of evidence have confirmed that Cel9A possesses the properties of both endo- and exocellulases [33]. A crystallographic study on a mutant composed of only the catalytic module of Cel9A and its CBM3c revealed that the CBM3c exhibits only poor binding properties to the cellulosic substrate but has a major role both for hydrolysis and processivity [34]. The recombinant form of this enzyme, *a*-9A, lacks the CBM2 and contains a dockerin instead. The integral CBM3c of the enzyme requires external targeting to the cellulosic substrate via other means, thus fulfilled by the scaffoldin-borne CBM3a, in order to function properly.

In addition, the overall activity of the enzymes was higher when the CBM was placed at the extremity of the scaffoldin (N or C terminus). This type of scaffoldin corresponds to the simple cellulosomal system, such as those of *C*. *cellulolyticum*, *C*. *cellulovorans*, *C*. *acetobutylicum* and *C*. *josui* [35–38]. Indeed, as *T*. *fusca* enzymes naturally occur in the free state, the position of the CBM at the termini of the scaffoldin may allow more freedom of movement [39].

In conclusion, this study is the first clear evidence that the position of the enzymes into the scaffoldin bears significance for optimization of cellulose degradation. It also demonstrates that the functionality of the enzymes is a critically important factor for enhanced synergistic activity. Finally, these properties appear suitable for a variety of bacterial systems and thus show that the selection of cohesin sequences in the design of chimaeric scaffoldins should no longer be neglected in future works involving designer cellulosomes.

## Supporting Information

S1 Fig Affinity-based ELISA binding assay of enzymes used in this study. For assay of the scaffoldins used in this study please see previous publication (Vazana et al. 2013). Enzymes were coated on ELISA plates at 1 μg/ml and interacted with relevant monovalent scaffoldins at 100 ng/ml. Primary antibody was against the CBM module.
(JPG)

S2 Fig Examples of non-denaturating PAGE gels between enzymes and scaffoldins to determine their precise stoichiometric concentrations. Lanes 1–7 correspond to complex of the specified enzyme and scaffoldin at different ratio: 0.4:1; 0.6:1; 0.8:1; 1:1: 1.2:1; 1.4:1; 1.6:1. Lanes 8 correspond to individual specified enzymes (100 pmol) and Lanes 9 correspond to individual specified scaffoldins (100 pmol). For all gels the amount of scaffoldins was fixed at 100 pmol, and the amount of enzymes was varied respective to the specified ratios (i.e., 1 is 100 pmol; 0.4 is 40 pmol etc.). The red rectangle shows the correct ratio at which both proteins are in exact equimolar amount, where neither enzyme nor scaffoldin is in excess.
(JPG)

S3 Fig Retention of the designer cellulosome complex after incubation for 72 h at 50°C. Non-denaturating PAGE gels between 3 enzymes: *a*-9A, *b*-48A, 5A-*t*, the lone scaffoldin (Scaf 22), and the resultant designer cellulosome complex. The enzymes and the scaffoldin were allowed to interact for 2 h at 37°C in buffer (see Materials & Methods). The gel in A was run immediately after whereas for gel B, the individual proteins and the complex were incubated for 72 h at 50°C before running.
(JPG)
