# Supplementary figures and images for: Significance of Relative Position of Cellulases in Designer Cellulosomes for Optimized Cellulolysis
Source: PLoS One. 2015 May 29;10(5):e0127326. doi: 10.1371/journal.pone.0127326 (PMC4449128; doi:10.1371/journal.pone.0127326)

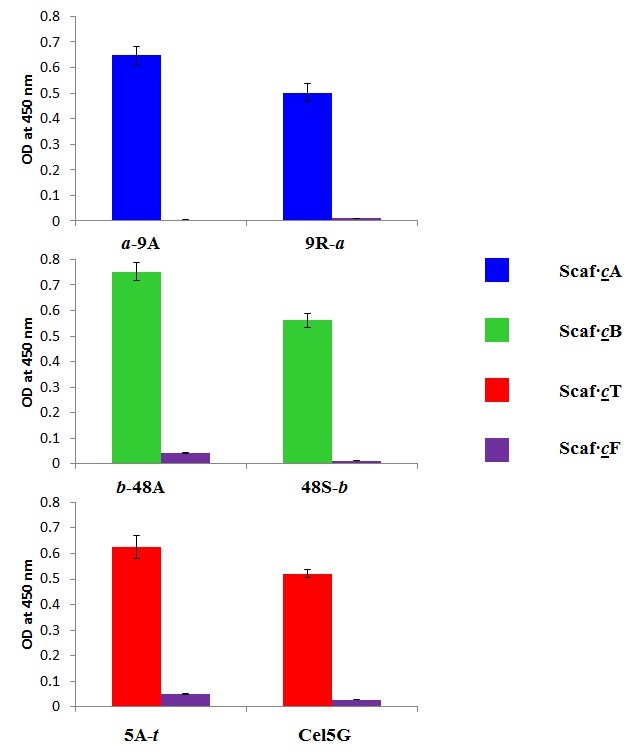

Supplement: S1 Fig — For assay of the scaffoldins used in this study please see previous publication (Vazana et al. 2013). Enzymes were coated on ELISA plates at 1 μg/ml and interacted with relevant monovalent scaffoldins at 100 ng/ml. Primary antibody was against the CBM module. (JPG) [file pone.0127326.s001.jpg]

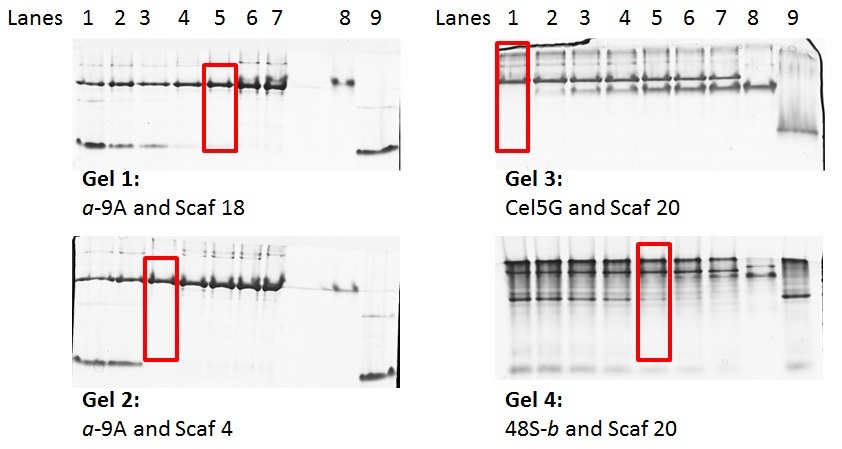

Supplement: S2 Fig — Lanes 1–7 correspond to complex of the specified enzyme and scaffoldin at different ratio: 0.4:1; 0.6:1; 0.8:1; 1:1: 1.2:1; 1.4:1; 1.6:1. Lanes 8 correspond to individual specified enzymes (100 pmol) and Lanes 9 correspond to individual specified scaffoldins (100 pmol). For all gels the amount of scaffoldins was fixed at 100 pmol, and the amount of enzymes was varied respective to the specified ratios (i.e., 1 is 100 pmol; 0.4 is 40 pmol etc.). The red rectangle shows the correct ratio at which both proteins are in exact equimolar amount, where neither enzyme nor scaffoldin is in excess. (JPG) [file pone.0127326.s002.jpg]

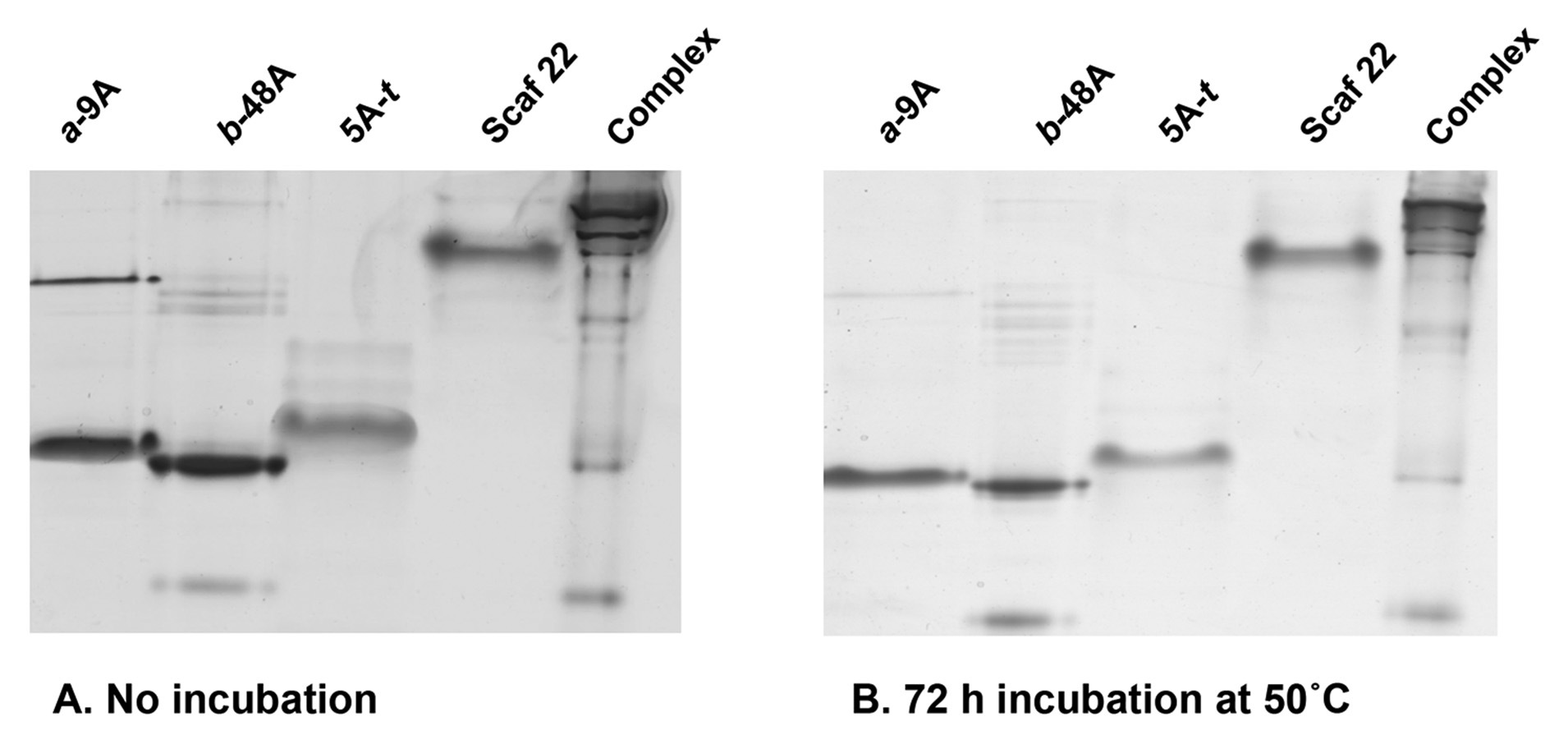

Supplement: S3 Fig — Non-denaturating PAGE gels between 3 enzymes: a-9A, b-48A, 5A-t, the lone scaffoldin (Scaf 22), and the resultant designer cellulosome complex. The enzymes and the scaffoldin were allowed to interact for 2 h at 37°C in buffer (see Materials & Methods). The gel in A was run immediately after whereas for gel B, the individual proteins and the complex were incubated for 72 h at 50°C before running. (JPG) [file pone.0127326.s003.jpg]
